# Major components of metabolic syndrome and nutritional intakes in different genotype of UCP2 −866G/A gene polymorphisms in patients with NAFLD

**DOI:** 10.1186/s12967-016-0936-3

**Published:** 2016-06-14

**Authors:** Mahdieh Abbasalizad Farhangi, Fatemeh Mohseni, Safar Farajnia, Mohammad-Asghari Jafarabadi

**Affiliations:** Nutrition Research Center, Department of Community Nutrition, School of Nutrition, Tabriz University of Medical Sciences, Tabriz, Iran; Drug Applied Research Center (DARC), Tabriz University of Medical Sciences, Tabriz, Iran; Road Traffic Injury Research Center, Tabriz University of Medical Sciences, Tabriz, Iran

**Keywords:** Uncoupling protein 2 (UCP2) gene, −866G/A polymorphism, Nonalcoholic fatty liver disease (NAFLD), Nutrition

## Abstract

**Background:**

It has been suggested that dietary modifications in combination with genetic predisposition play an important role in the pathogenesis of NAFLD. In the current study we aimed to investigate the major components of metabolic syndrome in patients with non-alcoholic fatty liver disease (NAFLD) and nutritional intakes according to different genotype of uncoupling protein-2 (UCP2) −866G/A gene polymorphism in these patients.

**Methods:**

In this study 151 participants including 75 patients with NAFLD and 76 healthy individuals were enrolled. Dietary intakes were assessed using a semi-quantitative food-frequency questionnaire. Physical activity was obtained by metabolic equivalent questionnaire. Anthropometric assessments were conducted by a trained researcher and body mass index and waist to hip ratio were calculated. Body composition was measured by bioelectrical impedance analysis and biochemical assays including fasting serum glucose, liver enzymes and lipid profiles were measured. Polymorphisms of −866G/A UCP2 gene was determined using polymerase chain reaction-restriction fragment length polymorphism method.

**Results:**

Serum triglyceride concentrations in 53.3 % of NAFLD patients compared with 35.5 % of control group was more than 150 mg/dl (*P* = 0.034). A significantly higher prevalence of low serum high density lipoprotein cholesterol concentrations was also observed in female NAFLD patients (*P* < 0.001). Dietary intakes in NAFLD group were not significantly different compared with control group (*P* > 0.05). However, according to genotypes patients with AG genotype had significantly higher protein consumption compared with control group (*P* < 0.05). Significantly higher consumption of dietary iron and copper in NAFLD patients with AG genotype was only observed among patients with NAFLD. However, the comparison of macro and micronutrient intakes in control group sound for stronger differences for AA genotype although these differences did not achieve significant threshold.

**Conclusions:**

A high prevalence of metabolic abnormalities was reported among NAFLD patients. Additionally, among NAFLD group, patients with AG genotype significantly consumed more protein, iron and copper in their usual diet.

## Background

Nonalcoholic fatty liver disease (NAFLD) is defined as abnormal lipid deposition in the hepatocytes in the absence of significant amount of alcohol intake. NAFLD includes nonalcoholic steatohepatitis (NASH) associated with markedly increased risk of cardiovascular and liver-related mortality which finally progress to more severe forms of liver disease such as advanced fibrosis, cirrhosis and even hepatocellular carcinoma [1, 2].

In developed countries, the prevalence of the NAFLD is up to 30 % in the general population, 50 % in patients with type 2 diabetes mellitus (T2DM), 76 % in obese people and almost 100 % in patients with morbid obesity [3, 4]. According to data from the US National Health and Nutrition Examination Survey (NHANES) report, the prevalence of NAFLD has increased from 47 to 75 % between 1988 and 2008 [5, 6]. In a large population-based study in southern regions of Iran in 2011, 21.5 % the prevalence of NAFLD in the adult general population was reported [5].

In addition to genetic predisposition, change in lifestyles and dietary habits increases the prevalence of obesity, diabetes mellitus, metabolic syndrome, cardiovascular disease and their consequences such as NAFLD throughout the world [7–9]. The long-term excessive food intake and dietary composition in food groups, macronutrients and micronutrients is associated with progression of NAFLD mostly recognized by abnormal ultrasonography (US) findings or elevated alanine aminotransferase (ALT) and aspartate aminotransferase (AST) concentrations as markers of liver injury [10, 11]. In general, lower antioxidant consumption, higher intake of calorie, carbohydrate, protein and high dietary cholesterol stimulate hepatic lipid accumulation leading to development of fatty liver disease [12–14]. In addition, inadequate intake of micronutrients, copper and iron may also be involved in pathogenesis of NAFLD [15]. NAFLD symptoms manifested by abnormalities in serum and hepatic stores of copper and iron in rodent models [16, 17] and low hepatic copper levels is known to be correlated with NAFLD progression and components of metabolic syndrome [16, 17]. However no report of the association between dietary intakes of these micronutrients and different genotypes of UCP2 in NAFLD is available. Additionally, oxidative stress has been involved in the pathogenesis of NAFLD; vitamin C and E are well-known antioxidants capable in blocking distribution of radical reactions [18]. These antioxidants play important roles in histological improvement of inflammation in NAFLD [18, 19].

Dietary recommendation should be appropriate according to an individual status and even genetic background [20]. Several studies mostly in animal models introduced diet as a potent modifier of NAFLD-related genes expression [10]; for example enhanced ω-6/ω-3 poly unsaturated fatty acids (PUFAs) ratio interacts with PNPLA3 rs738409 gene in the GG homozygote and enhances ALT concentrations and hepatic fat accumulation in human [21]. Other studies also revealed the role of ω-3 fatty acids as regulators of hepatic gene expression by mainly aiming the transcription factors sterol regulatory element binding transcription factor 1 (SREBP-1c) and down-regulating inflammatory genes [22].

Three common polymorphisms in uncoupling protein 2 (UCP2) are −866G>A (rs659366), 55 Ala/Val (rs660339) and 3-UTR ins > del [23]. The relationship of −866G>A gene polymorphism of UCP2 (rs659366) with obesity and type 2 diabetes has been reported previously [23]. UCP2 is located in the inner membrane of mitochondria, acts as a mediator of proton leak and ultimately leads to decreased ATP production and energy release [24]. The wide tissue distribution of UCP2 shows its potent role in several pathologic events on specific tissue or organs [25, 26]. Enhanced UCP2 expression is able to respond oxidative stress by controlling production of mitochondrial superoxide [26]; therefore, it may be a therapeutic target for management of oxidative damage and metabolic imbalance in NAFLD [25].

Considering the lack of knowledge about the interaction between UCP2 gene polymorphism and nutrient intakes in NAFLD patients we aimed to investigate the interaction between energy and nutrients intake and −866G>A gene polymorphism of uncoupling protein 2 (UCP2) in patients with NAFLD.

## Methods

The present case control study was conducted among 75 patients with NAFLD and 76 healthy individuals; these two groups were matched by age and gender with group matching. Patients were recruited from the out patients gastroenterology clinics of Tabriz University of Medical Sciences. Disease diagnosis was confirmed by a physician based on the findings of ultrasonography (US). Written informed consent was obtained from all of subjects before participation in the study. The protocol of the study has been approved by the ethics committee of Tabriz University of Medical Science (Registration Code: 11013).

Inclusion criteria were as follows: aged between 20 and 50 years old, BMI between 25 and 39.9 kg/m^2^. The exclusion criteria were: any history of acute or chronic liver diseases, viral hepatitis, hemochromatosis, Wilson’s disease, autoimmune or endocrine disorders, pregnancy or lactation, alcohol consumption, using hepatotoxic medications including corticosteroids, amiodarone, valproate and being on weight loss diets for at least 3 months prior participation in study.

### Anthropometric assessments

Weight was measured with a calibrated scale (SECA, Hamburg, Germany) to the nearest 0.1 kg with the minimal clothing without shoes and height using a non-stretchable measurement tape with the precision of 0.1 cm. The measurements were taken one time by a trained researcher. The body mass index (BMI) was calculated as weight (kg) divided by height squared (m^2^). Waist circumference (WC) was measured in standing position at the level of the umbilicus and hip circumference (HC) was measured at the maximum point between the hip and the buttock with a non-elastic tape.

### Physical activity level

Physical activity was obtained by the questionnaire with nine different metabolic equivalent (MET) scales ranging from sleep/rest (0.9 METs) to high-intensity physical activities (>6 METs) [27]. This questionnaire has been validated among Iranian people [28] and evaluates usual daily physical activity by simple self-reported values. The precision of this tool is comparable with the findings of international physical activity questionnaire (IPAQ) [27].

For each activity level, the MET value was multiplied by the time spent at that particular level. The MET-time at each level was added to obtain a total over 24 h MET-time, representing the physical activity level on an average weekday. Physical activities of different intensities were categorized to sedentary (<3 METs), moderate (3–6 METs) and vigorous (>6 METs) respectively [27].

### Biochemical assessments

After an overnight fasting, all of patients underwent a laboratory examination. Venous blood samples were taken from individuals and approximately 2 cc of the blood was transferred into tubes containing ethylene di-amine tetra acetic acid (EDTA) for genetic assays. Sera were also extracted from remaining blood samples for biochemical assays including fasting serum glucose (FSG), ALT, AST, total cholesterol (TC), triglyceride (TG) and high density lipoprotein cholesterol (HDL-C). Laboratory assessments were performed by Abbott ALCYON™ 300 auto analyzer using commercial ELISA kits (Pars-Azmoon, Tehran, Iran). All of the biochemical assays were performed by a trained lab assistant who was blinded to group assignments. Serum low density lipoprotein cholesterol (LDL-C) was calculated by Friedewald formula: LDL-C = TC − (HDL-C − TG/5) [29].

Biochemical parameters were also categorized according to the National Cholesterol Education Program’s Adult Treatment Panel III report (NCEP-ATP III) criteria [30] (except for waist circumference which was defined as ≥90 cm for both genders for Iranian population) for defining metabolic abnormalities [31, 32].

### Dietary intake

Dietary intakes were assessed using a semi-quantitative food-frequency questionnaire (FFQ) adapted to the Iranian society [33]. Since the efficiency of a FFQ is related to the culture and ethnic background of the study population, the validity and reliability should be conducted in different population [34]. The FFQ included 168 food items with specified serving sizes commonly consumed by Iranians. Participants reported their average frequency intake of each food item in terms of the number of specified serving sizes consumed per day/week/month/year, or never. The reported frequency of consumed foods and portion sizes for each food item were converted to a daily intake.

### Body composition analysis

Fat mass (FM) and fat free mass (FFM) were determined using a Bioelectrical Impedance Analysis (BIA; Tanita BC-418, Tanita Corp., Tokyo, Japan). Analysis were carried out for all subjects after 12 h of fasting period while individuals wore light clothes, no shoes, no jewellery and no heavy physical activity and alcohol consumption or diuretic intake prior to analysis [35].

### DNA extraction and genotyping

**Fig. 1:**
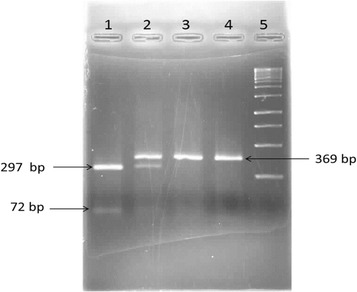
PCR-RFLP analysis for UCP2 −866G/A polymorphism. *Lane 1* GG genotype. *Lane 2* AG genotype. *Lane 3* AA genotype. *Lane 4* Undigested PCR product. *Lane 5* DNA size marker

Genomic DNA was extracted from the blood cells by salting out method [36]. This technique is a simple deproteinization cell procedure by dehydration and precipitation with saturated NaCl solution. Single nucleotide gene polymorphism (SNP) was detected by polymerase chain reaction-restriction fragment length polymorphisms (PCR-RFLP) method. DNA fragment analogous to −866 A/G polymorphism (rs659366) in UCP2 gene was amplified by 5′-CACGCTGCTTCTGCCAGGAC-3′ as forward primer and 5′-AGGCGTCAGGAGATGGACCG-3′ and a reverse primer. 1µ Genomic DNA in addition to 0.2µ Taq DNA polymerase and 1µ of each primer were added to 22µ of 1× PCR master-mix. PCR conditions were: a primary denaturation at 95 °C for 5 min followed by 38 cycles of denaturation at 94 °C for 1 min; annealing at 59 °C for1 min; extension at 72 °C for 46 s and a final extension at 72 °C for 5 min. The PCR products were run for visualization on 1 % agarose gel and stained by ethidiumbromide. 10µ of PCR products were digested by 0.3µ *MLU*I restriction enzyme, incubated at 37 °C for 1.5 h and ultimately separated on 2 % agarose gel electrophoresis. The (−866) AA genotype was indicated by a single 369 base-pair (bp) fragment as result of loss of *Mlu*I site, whereas, the wild-type (−866) GG genotype was digested into 297 and 72 bp fragments (Fig. 1).

### Statistical methods

The SPSS statistical software package version 16 (SPSS Inc., Chicago, IL, USA) was used for all statistical analyses. Kolmogorov–Smirnov test was performed for normality of the distributions of variables. Demographic characteristics, energy intake and nutrients intake of the two groups were compared by means of analysis of co-variance (ANCOVA) and one way ANOVA tests. The Chi Square test and student’s *t* test were used for comparison of categorical and continuous variables between groups respectively. P values lower than 0.05 has been considered to be statistically significant.

## Results

The demographic, anthropometric and biochemical parameters of the study population are presented in Table 1. No significant difference was observed in mean age, BMI and fat free mass between NAFLD and control groups; However, WC and WHR in NAFLD patients were significantly higher compared with healthy group (*P* < 0.05). Among biochemical variables, serum HDL-C and LDL-C concentrations were significantly lower and serum AST, ALT and TG concentrations were significantly higher in NAFLD patients compared with control group (*P* < 0.05 and *P* < 0.01 respectively). Table 2 presents the prevalence of several components of metabolic syndrome according to ATP III criteria; significantly higher prevalence of WC ≥ 90 cm was observed in patients with NAFLD compared with control group (*P* = 0.037). Approximately 53.3 % of NAFLD patients compared with 35.5 % of control group had TG ≥ 150 mg/dl (*P* = 0.034). A significantly higher prevalence of low serum HDL-C concentrations was also observed in female NAFLD patients (*P* = 0.001).

**Table 1:** General characteristics of study subjects

Variable	NAFLD (N = 75)	Control (N = 76)	Mean difference (95 % CI)	P*
Sex				
Male [n (%)]	36 (48 %)	29 (38.2 %)	–	0.25
Female [n (%)]	39 (52 %)	47 (61.8 %)	–	
Age (years)	40.65 (8.41)	38.87 (8.2)	1.78 (−0.89 to 4.46)	0.18
BMI (kg/m^2^)	31.78 (4.17)	31.38 (4.04)	0.40 (−0.92 to 1.72)	0.54
WC (cm)	103.12 (9.46)	100.14 (8.72)	2.98 (0.55 to 5.90)	*0.04*
WHR	0.92 (0.06)	0.89 (0.06)	0.02 (0.002 to 0.04)	*0.03*
BMR (kcal/day)	1739.64 (290.53)	1679 (96.31)	60.64 (−33.74 to 155.02)	0.20
FM (%)	32.67 (8.58)	34.57 (8.02)	−1.90 (−4.57 to 0.76)	0.16
FFM (%)	67.06 (8.11)	65.51 (7.84)	−1.55 (−4.11 to 1.02)	0.23
TC (mg/dl)	183.44 (36.91)	187.96 (28.89)	−4.52 (−15.17 to 6.13)	0.40
HDL-C (mg/dl)	43.24 (11.4)	48.29 (11.6)	−5.05 (−8.75 to −1.35)	*0.008*
LDL-C (mg/dl)	104.11 (34.62)	111.52 (26.43)	−6.81 (−16.71 to 3.09)	*0.03*
FSG (mg/dl)	90.59 (11.24)	89.59 (9.93)	0.63 (−2.78 to 4.03)	0.71
ALT (IU/l)	49.96 (25.958)	26.84 (9.814)	23.12 (16.82 to 29.41)	<*0.001*
AST (IU/l)	32.99 (14.86)	23.08 (6.12)	9.91 (6.26 to 13.55)	<*0.001*
TG (mg/dl)	152.00 (114.00–225.00)	118.50 (79.50–198.00)	–	*0.004*
Physical activity				0.56
MET: 3–6	62 (82.7 %)	60 (78.9 %)	–	
MET > 6	13 (17.3 %)	16 (21.1 %)	–	

Italic values are statistically significant at P <0.05

*BMI* body mass index, *WC* waist circumference, *WHR* waist to hip ratio, *BMR* basal metabolic rate, *FM* fat mass, *FFM* fat free mass, *FSG* fasting serum glucose, *TC* total cholesterol, *TG* triglyceride, *HDL*-*C* high density cholesterol, *LDL*-*C* low density cholesterol, *ALT* alanine amino transferase, *AST* aspartate amino transferase, *MET* metabolic equivalent

* P value for sex, physical activity and TG based on Chi Square tests and *U* Mann–Whitney respectively; otherwise based on independent *t* test using equal variable. TG are presented based on median (percentile 25th–percentile 75th) and other variables data are presented based on mean (SD)

**Table 2:** The prevalence of several components of metabolic syndrome according to the National Cholesterol Education Program’s Adult Treatment Panel (ATP) III criteria [30, 31]

Variable	NAFLD (N = 75) [n (%)]	Control (N = 76) [n (%)]	P*
WC (cm) ≥ 90 cm	71 (94.7)	64 (84.2)	*0.03*
FSG (mg/dl) ≥ 110 mg/dl	2 (2.7)	1 (1.3)	0.55
TG (mg/dl) ≥ 150 mg/dl	40 (53.3)	27 (35.5)	*0.03*
HDL-C (mg/dl) < 40 mg/dl (male)	18 (50)	13 (44.8)	0.08
HDL-C (mg/dl) < 50 mg/dl (female)	24 (61.5)	18 (38.3)	*0.001*

Italic values are statistically significant at P <0.05

*WC* waist circumference, *FSG* fasting serum glucose, *TG* triglyceride, *HDL*-*C* high density cholesterol

* Based on Chi Square test

Mean energy and protein intake in NAFLD group was higher and vitamin C and E intake were lower in NAFLD patients compared with control group. However statistical significance was not achieved (Table 3).

**Table 3:** Comparison of energy, macro and micronutrient intakes between study groups

	NAFLD (N = 75)	Control (N = 76)	Mean difference (95 % CI)	*P*
Calories (kcal)	2815.06 (536.35)	2794.93 (448.64)	−20.13 (−179.29 to 139.03)	0.80
Protein (g/day)	93.88 (21.62)	88.88 (16.60)	−4.99 (−11.20 to 1.20)	0.11
Carbohydrate (g/day)	437.38 (89.72)	438.49 (79.58)	1.10 (−26.18 to 28.39)	0.93
Total fat (g/day)	89.70 (27.03)	91.52 (24.35)	1.82 (−6.45 to 10.10)	0.66
Vitamin E (mg/day)	14.08 (5.60)	14.63 (4.11)	0.80 (−1.03 to 2.12)	0.49
Vitamin C (mg/day)	124.18 (83.70–165.06)	116.71 (90.06–177.29)	–	0.84
Iron (mg/day)	23.37 (6.04)	22.86 (5.04)	0.90 (−2.30 to 1.27)	0.56
Copper (mg/day)	2.29 (0.48)	2.30 (0.53)	−0.004 (−0.16 to 0.16)	0.95

All of data are presented as mean (SD) except for the Vitamin C intake which is presented as median (percentile 25th–percentile 75th). P value of Vitamin E and C based on U Mann–Whitney and for other variables as independent sample *t* test

The comparison of dietary intakes according to −866A/G of UCP2 gene polymorphism between case and control groups is presented in Table 4. Patients in AG genotype have significantly higher protein consumption compared with control group (*P* < 0.05). Also patients in this genotype have significantly higher carbohydrate, iron and copper consumption in comparison of patients in other genotypes (*P* < 0.05). No significant difference was observed for other nutrients.

**Table 4:** The comparison of energy, macro and micronutrient intakes according to −866A/G of UCP2 gene polymorphism between study groups

Variable	Genotype	NAFLD (N = 75)	Control (N = 76)	Mean difference (95 % CI)	*P* ^†^
Calories (kcal/day)	*AA*	2674.88 (774.39)	3089.64 (342.12)	−431.75 (−2515.28 to 1651.79)	0.61
	*AG*	2920.76 (496.14)	2852.15 (425.28)	6.23 (−200.83 to 213.28)	0.95
	*GG*	2672.68 (534.37)	2708.95 (467.26)	−29.76 (−241.50 to 181.97)	0.78
*P* ^‡^		0.14	0.16		
Fat (g/day)	*AA*	96.22 (46.68)	96.67 (15.87)	−12.64 (−127.33 to 102.04)	0.78
	*AG*	88.94 (24.24)	94.51 (23.63)	−3.89 (−15.25 to 7.46)	0.49
	*GG*	89.69 (28.02)	88.13 (25.75)	1.75 (−11.48 to 14.99)	0.79
*P* ^‡^		0.85	0.49		
Protein (g/day)	*AA*	84.96 (22.77)	99.80 (15.56)	1.050 (−59.55 to 61.65)	0.96
	*AG*	98.67 (20.62)	87.77 (15.54)	7.55 (−0.13 to 15.22)	*0.04*
	*GG*	87.90 (21.74)	88.75 (17.62)	−0.60 (−8.71 to 7.50)	0.88
*P* ^‡^		0.07	0.39		
Carbohydrate (g/day)	*AA*	398.97 (111.25)	476.35 (53.66)	−73.33 (−373.72 to 227.06)	0.55
	*AG*	460.26 (80.22)	455.29 (92.83)	−13.55 (−50.62 to 23.51)	0.46
	*GG*	408.06 (92.58)	418.51 (63.02)	−9.61 (−42.35 to 23.13)	0.55
*P* ^‡^		*0.03*	0.09		
Iron (mg/day)	*AA*	20.44 (4.34)	24.812 (4.11)	−2.58 (−16.33 to 11.16)	0.64
	*AG*	24.93 (6.48)	23.10 (5.49)	0.48 (−2.06 to 3.02)	0.70
	*GG*	21.44 (4.86)	22.41 (4.72)	−0.93 (−3.05 to 1.19)	0.38
*P* ^‡^		*0.03*	0.62		
Copper (mg/day)	*AA*	2.05 (0.55)	2.37 (0.29)	−0.19 (−1.70 to 1.31)	0.75
	*AG*	2.44 (0.53)	2.37 (0.56)	−0.05 (−0.28 to 0.18)	0.64
	*GG*	2.12 (0.50)	2.22 (0.40)	−0.09 (−0.30 to 0.12)	0.39
*P* ^‡^		*0.03*	0.39		
Vitamin E (mg/day)	*AA*	12.67 (8.53–21.93)	16.79 (13.69–22.42)	–	0.22
	*AG*	13.00 (10.09–17.18)	14.70 (11.30–17.82)	–	0.34
	*GG*	11.67 (9.63–17.54)	14.04 (11.24–17.97)	–	0.33
*P* ^‡^		0.96	0.29		
Vitamin C (mg/day)	*AA*	81.92 (47.27–249.34)	118.56 (90.91–151.10)	–	0.14
	*AG*	126.91 (95.77–178.86)	103.65 (83.98–184.27)	–	0.51
	*GG*	123.22 (73.77–137.46)	121.90 (94.37–163.71)	–	0.39
*P* ^‡^		0.07	0.99		

Italic values are statistically significant at P <0.05

Vitamin C intake is presented as median (percentile 25th–percentile 75th) and other as mean (SD)

^†^P-value for analysis of co variance (ANCOVA) adjusted for confounding effects of age and gender

^‡^P value of vitamins E and C based on Kruskal–Wallis and otherwise based on one way ANOVA

## Discussion

In the current case–control study, we demonstrated a higher dietary intake of protein, carbohydrate, iron and copper in the AG genotype of UCP2 gene polymorphisms in patients with NAFLD. These patients had also higher values of central obesity indices and metabolic abnormalities compared with healthy individuals. In our review of the literature, this is the first report evaluating the association between 866A/G UCP2 gene polymorphism and nutritional intakes in patients with NAFLD. Our results showed a higher intake of energy and protein among NAFLD patients compared with healthy control subjects.

Consistent with our findings, Zelber-Sagi et al. [37] and Silva et al. [38] reported higher consumption of energy and protein in patients with NAFLD. Hashemi-Khani et al. [12] reported a significantly higher amount of carbohydrate and fat intake and no significant higher consumption of protein among one hundred NAFLD patients compared with healthy individuals. Interestingly they also reported a lower nutrient adequacy ratio (NAR) as an index of dietary quality in these patients (*P* < 0.05). We did not achieve significant threshold in our energy and nutrient intakes values probably because of our lower sample size or difference in disease stage compared with these studies. The higher energy and carbohydrate intake would finally lead to anthropometric and metabolic abnormalities as confirmed in the current study; waist circumference as an index of central obesity and metabolic syndrome [39] was higher in our NAFLD group. Also higher serum aminotransferase concentrations and a significantly high prevalence of metabolic syndrome components including elevated serum TG and low serum HDL-C concentrations were also reported in our NAFLD patients. In Clark et al. study [40] there was a significant association between high aminotransferase and WC and higher BMI in NAFLD patients. Rocha et al. [2] also reported a strong association between high WC and components of metabolic syndrome in patients with NAFLD (*P* < 0.001). Elevated serum aminotransferase concentrations have been associated with general and central obesity indices and elevated triglyceride concentrations in these patients [2, 40].

Interestingly, we reported a significantly higher consumption of dietary iron and copper in AG genotype of UCP2 gene polymorphisms in patients with NAFLD. This difference was only observed among patients with NAFLD and not in control group. However, the comparison of macro and micronutrient intakes in control group sounds for stronger differences for AA genotype. This finding further confirms the possible role of AG genotype–nutrient interaction in pathogenesis NAFLD.

Similar finding was also reported by Silva et al. [38]; however unlike our study, no interaction between dietary intakes and gene polymorphisms has been evaluated in their study. Higher dietary iron consumption and especially heme–iron may play a role in the pathogenesis of NAFLD by increasing oxidative stress [41, 42]. Iwasa et al. [43] and Yamamoto et al. [44] have demonstrated that lower intakes of calorie and iron lead to reduce visceral fat, liver enzymes and ferritin in patients with chronic liver disease and nonalcoholic fatty disease.

It seems that AG genotype of UCP2 is an important genetic predisposing factor of metabolic disease including obesity and T2DM [24, 45, 46]; in parallel of these observations, our study is the first study reported the gene-nutrient interactions between −866 A/G of UCP2 gene polymorphism and NAFLD.

Our NAFLD participants had non-significantly lower intake of antioxidants including vitamins C and E. Previous reports also highlighted the poor antioxidant intakes in these patients; Musso et al. [47] reported a low dietary ascorbic acid and tocopherol intake in patients with non-alcoholic steato-hepatitis. These findings were also been supported in the study of Erhardt et al. [48] reporting reduced tocopherol and carotenoid consumption as dietary antioxidants compounds in patients with NASH compared to healthy group. The non-significance threshold of our findings might be attributed to the difference in the stage of disease compared with these two studies.

## Conclusions

The present study reported a high prevalence of metabolic abnormalities in NAFLD patients. In addition, NAFLD patients with AG genotype consumed more calories, protein, carbohydrate, iron and copper and low amounts of vitamin C and E in their usual diet. However, statistical significant differences were only observed in protein, iron and copper consumption among NAFLD group. It is clear that dietary modification is the easiest and even the most efficient way to reduce chronic disease risk factors [49, 50]. This is the first study comparing nutritional intakes according to UCP2 −866G/A gene polymorphism in patients with NAFLD; however several limitations of the current study should also be addressed; first, the case control design of the study has not potential to address cause and effect relationship between variables. Second, we did not evaluate the markers of oxidative stress in our study. Further studies with larger sample size and interventional designs are needed to determine the association of dietary compounds and different UCP2 genotypes in nonalcoholic fatty liver disease.

## Abbreviations

ALT: alanine aminotransferase
ANCOVA: analysis of co-variance
AST: aspartate aminotransferase
BMI: body mass index
BIA: bioelectrical impedance analysis
FFM: fat free mass
FM: fat mass
FFQ: food frequency questionnaire
HC: hip circumference
HDL-C: high density lipoprotein cholesterol
IPAQ: international physical activity questionnaire
LDL-C: low density lipoprotein cholesterol
MET: metabolic equivalent
NAFLD: non-alcoholic fatty liver disease
NASH: nonalcoholic steatohepatitis
NHANES: National Health and Nutrition Examination Survey
SNP: single nucleotide gene polymorphism
TC: total cholesterol
TG: triglyceride
T2DM: type 2 diabetes mellitus
UCP: uncoupling protein
US: ultrasonography
WC: waist circumference
WHR: waist to hip ratio
